# “A Delicate balance”—Perceptions and Experiences of ICU Physicians and Nurses Regarding Controlled Donation After Circulatory Death. A Qualitative Study

**DOI:** 10.3389/ti.2022.10648

**Published:** 2022-09-06

**Authors:** Matthieu Le Dorze, Sara Martouzet, Etienne Cassiani-Ingoni, France Roussin, Alexandre Mebazaa, Lucas Morin, Nancy Kentish-Barnes

**Affiliations:** ^1^ AP-HP, Hôpital Lariboisière, Department of Anesthesia and Critical Care Medicine, Paris, France; ^2^ Université Paris-Saclay, UVSQ, INSERM, CESP, U1018, Villejuif, France; ^3^ Université de Tours, EA 7505 Éducation, Éthique et Santé, Tours, France; ^4^ Université de Paris, Inserm, UMRS 942 Mascot, Paris, France; ^5^ INSERM CIC 1431, University Hospital of Besançon, Besançon, France; ^6^ AP-HP, Saint Louis University Hospital, Famiréa Research Group, Medical Intensive Care Unit, Paris, France

**Keywords:** organ donation, qualitative research, end of life, controlled donation after circulatory death, withdrawal of life-sustaining treatments

## Abstract

Controlled donation after circulatory death (cDCD) is considered by many as a potential response to the scarcity of donor organs. However, healthcare professionals may feel uncomfortable as end-of-life care and organ donation overlap in cDCD, creating a potential barrier to its development. The aim of this qualitative study was to gain insight on the perceptions and experiences of intensive care units (ICU) physicians and nurses regarding cDCD. We used thematic analysis of in-depth semi-structured interviews and 6-month field observation in a large teaching hospital. 17 staff members (8 physicians and 9 nurses) participated in the study. Analysis showed a gap between ethical principles and routine clinical practice, with a delicate balance between end-of-life care and organ donation. This tension arises at three critical moments: during the decision-making process leading to the withdrawal of life-sustaining treatments (LST), during the period between the decision to withdraw LST and its actual implementation, and during the dying and death process. Our findings shed light on the strategies developed by healthcare professionals to solve these ethical tensions and to cope with the emotional ambiguities. cDCD implementation in routine practice requires a shared understanding of the tradeoff between end-of-life care and organ donation within ICU.

## Graphical Abstract

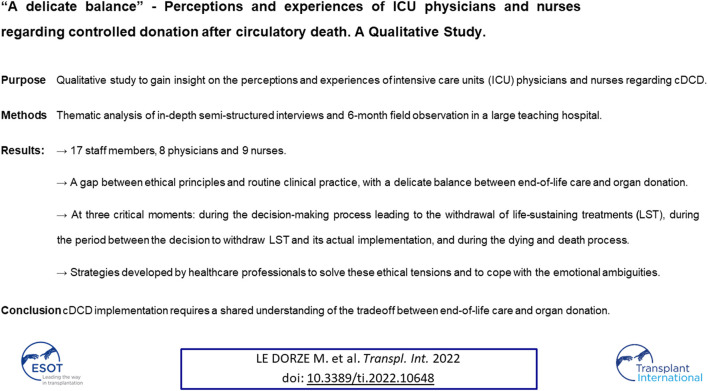

## Introduction

Controlled donation after circulatory death (cDCD) refers to organ donation from patients whose death is defined using circulatory criteria after the planned withdrawal of life-sustaining treatments (WLST) (1). The scarcity of donor organs and the good transplantation outcomes (2–4) legitimately support the development of this type of donation (5–7) in a context where WLST decisions occur more and more frequently in intensive care units (ICU) worldwide (8–10).

cDCD reshapes end-of-life care by introducing the issue of organ donation before the time of death. Thus, cDCD may potentially affect not only the decision-making process leading to WLST but also other end-of-life care practices (11–14). The French cDCD protocol explicitly states that decision to withdraw LST must be made in the patient’s best interest, independently from any consideration regarding organ donation, and that cDCD must not alter end-of-life care (15). Yet, healthcare professionals can feel particularly uncomfortable when, in practice, end-of-life care and organ donation overlap (16–18). The challenge is not only to identify potential cDCD donors, but also to give healthcare professionals a reassuring ethical framework. Research has shown that physicians and nurses working in ICUs are not always at ease with organ donation after brain death (19).

Developing knowledge on the perceptions and experiences of healthcare professionals regarding cDCD is crucial to improve the quality of the process but remains rarely investigated (16, 17, 18, 20, 21). We conducted a cross-sectional qualitative study to better understand ICU physicians’ and nurses’ experience of cDCD. This will enable to develop interventions to support and guide them throughout this practice, which in turn should not only improve their experience but also the experience of patients’ relatives.

## Materials and Methods

To carry out this monocentric qualitative study in an optimal way, we brought together a multidisciplinary research team, which included an ICU physician involved in organ donation (MLD), a graduate student in anthropology student (SM), and a sociologist (NKB).

### Objectives

Our objectives were to understand how healthcare professionals perceived WLST decision-making process and how they experienced end-of-life care in this particular context, and finally how their relationship with the patient’s relatives was affected**.**

### Design

#### In-Depth Interviews

Between May and November 2019, we conducted in-depth interviews with healthcare professionals working in the ICU of a large teaching hospital in central Paris (610 beds overall, including 29 ICU beds). In this ICU, cDCD is implemented according to the ethical and technical requirements of the nationwide protocol, particularly with the systematic use of normothermic regional perfusion (15). The WLST take place preferentially in the ICU, which facilitates the support of relatives by clinicians. When lung retrieval is considered, WLST is exceptionally done in the operating room. In all cases, the ICU team takes care of the patient until death and presence of family members is encouraged if they wish. After the declaration of death, the organ procurement team and a surgical team collaborate on the cannulation and the start of the normothermic regional perfusion.

The semi-structured interview guide was developed *a priori* by the investigators (Supplementary Table S1). Questions were open-ended, which allowed participants to describe their experience in their own words and to broach specific issues that they considered relevant.

#### Field Observation

In addition, one investigator (SM) immersed herself full-time in the ICU for a 6-month field observation to better understand the professional culture and the institutional context in which the interviews were conducted (22).

### Data Collection

#### In-Depth Interviews

We used purposeful sampling based on professional status (physicians/nurses) and number of cDCD experiences (23). Participants were recruited through e-mail and personal solicitations. Interviews were conducted individually and in-person by a single investigator (SM) and lasted between 1 and 2 h. All interviews were audio recorded, pseudonymized, and then transcribed verbatim for analysis. Data collection was interrupted when we reached data saturation, namely when no new themes emerged from the interviews (24).

#### Field Observation

Detailed descriptive notes were taken in the form of a daily research journal. Reflective field notes were also taken. These notes go beyond descriptions to include the researcher’s problems, impressions, analyses, clarifications, syntheses, connections, and other ideas about the research project.

### Data Analysis

#### Primary Data, Interviews

Three researchers (MLD, NKB, and SM) read all the transcripts. Using an inductive approach, they identified initial key themes and concepts that occurred throughout the first three interviews using thematic analysis (25). Then they developed a codebook through an iterative process that ended when the three authors had achieved consensus (26). These authors then coded the same three interviews independently to check for intercoder reliability, after which they convened as a group to discuss potential disagreements and refine the initial themes and categories. Using this consolidated codebook, one researcher (SM) then coded the remaining interviews, adding or modifying codes as necessary given the content of subsequent interviews. Any difficulties or uncertainties were discussed with NKB and MLD during research meetings.

#### Secondary Data, Observation

Field notes were coded by SM and then discussed and analyzed by NKB and MLD. Field notes allowed us to develop a comprehensive and richer understanding of the interviews and helped confirm thematic analysis of interviews.

## Results

A total of 20 staff members were interviewed but due to saturation, a total of 17 were analyzed, including interviews with 8 physicians and 9 nurses (Table 1). No clinician approached refused an interview. Qualitative analysis highlighted the ethical tensions experienced by clinicians at different stages of the process. We identified three key phases in the process, each with specific tensions. These phases and their associated perceived ethical tensions are described below. For each phase, we derived a sample of representative quotes is provided in Tables 2, 3, 4.

**TABLE 1:** Characteristics of the study participants.

Code	Role	Age range	Sex	ICU experience	cDCD experience
P01	Senior physician	31–40 years	Man	5–10 years	5 to 10 procedures
P02	Senior physician	31–40 years	Man	5–10 years	5 to 10 procedures
P03	Senior physician	31–40 years	Man	10–15 years	>10 procedures
P04	Senior physician	41–50 years	Man	15–20 years	>10 procedures
P05	Senior physician	31–40 years	Man	5–10 years	5 to 10 procedures
P06	Senior physician	51–60 years	Man	>20 years	5 to 10 procedures
P07	Senior physician	31–40 years	Man	10–15 years	5 to 10 procedures
P08	Senior physician	31–40 years	Woman	5–10 years	1 to 5 procedures
N01	Nurse assistant	41–50 years	Woman	>20 years	5 to 10 procedures
N02	Nurse	21–30 years	Man	5–10 years	1 to 5 procedures
N03	Nurse	21–30 years	Woman	0–5 years	1 to 5 procedures
N04	Nurse	31–40 years	Woman	5–10 years	5 to 10 procedures
N05	Nurse	51–60 years	Man	>20 years	>10 procedures
N06	Nurse	31–40 years	Woman	5–10 years	5 to 10 procedures
N07	Nurse	21–30 years	Man	0–5 years	1 to 5 procedures
N08	Nurse	21–30 years	Woman	0–5 years	1 to 5 procedures
N09	Nurse	31–40 years	Man	10–15 years	5 to 10 procedures

**TABLE 2:** The decision-making process leading to the withdrawal of life-sustaining treatments in a context of potential organ donation Domains and Quotes.

A gap between theory and practice
Quote 1: “When we decide to withdraw life sustaining treatments, the intention is completely schizophrenic. We are told that the two processes must be totally sealed. In practice, this is impossible! All the doctors, everyone will tell you … it’s impossible to dissociate the two. It’s the same team who decides to withdraw life sustaining treatments and who calls the coordination office to start the organ procurement process. It’s rather hypocritical” (Physician interview P03)
Quote 2: “Of course there is porosity between the two. cDCD is something we have in mind before, and it is a difficulty” (Physician interview P01)
Formal and informal communication
Quote 3: “We know the patients who are potentially Maastricht 3 donors. We talk about it among ourselves, not in an official, written way, but we know that a decision to withdraw treatment can lead to a M3” (Nurse interview N06)
Quote 4: “The nursing staff attends the collegial procedure meetings. It’s extremely important for them that we make a clear and complete distinction between withdrawal of life sustaining treatments and Maastricht 3 organ donation process” (Physician interview P02)
Quote 5: “It’s important that everyone adheres to the project, it allows us to feel comfortable. In any case, that everyone is clear with the situation and that everyone has been able to express themselves. It’s very important that it goes well between us. Because if it all goes well, people will agree to do it again” (Physician interview P04)
Quote 6: “In practice, we are not going to delude ourselves: we tend to anticipate, at least among ourselves (physicians), the possibility of a cDCD” (Physician interview P05)
End-of-life care as a process, organ donation as a procedure
Quote 7: “End of life and Maastricht 3 are really dissociated. What’s most important is the patient’s end of life. Maastricht 3, when you understand that it’s just a procedure –and therefore it’s a technique and an organization – then it’s no longer a problem, in fact. What’s important is what is upstream” (Physician interview P04)
Making sense of the ethical dilemma
Quote 8: “There’s nothing more we can do, the patient is going to die, and it may save someone else’s life. I like this way of looking at things. I find that it de-dramatizes the situation. It breaks the tragic image of death. In the end, he didn’t die for nothing. It gives a meaning to death” (Nurse interview N06)
Quote 9: “There is a real social benefit behind the process and a true purpose for the recipients”( Physician interview P03)

**TABLE 3:** The period between the decision to withdraw life-sustaining treatment and its actual implementation. Domains and Quotes.

A difficult compromise between end-of-life care and organ preservation
Quote 1: “Do we resuscitate to preserve the organs, or do we let this patient die because there is no therapeutic plan?” We shouldn’t resuscitate someone who doesn’t have a therapeutic plan. It’s not clear at all. This time period is what we find the most disturbing; we know the patient is going to die but how far should we go to preserve his organs?“( Physician interview P07)
Quote 2: “I asked myself whether it is ethically acceptable to keep the patient alive for his organs”( Nurse interview N06)
Quote 3: “It’s really invasive, it may seem really aggressive, but I think it’s the right solution for organ preservation” (Nurse interview N05)
A time to support relatives
Quote 4: “You have to explain again and again, you have to try to be as clear and simple as possible, you have to make them understand that it will be long and difficult. It requires relational skills” (Nurse interview N08)
Quote 5: “As the family is here just waiting it gives us a little more time together. This is the moment to give them (the family) space, to give them as much time as possible with their loved one, and to give them time to accept the situation” (Nurse interview N04)
Quote 6: “It also gives us the opportunity to prepare the patient and to focus on the person in the bed” (Nurse interview N08)

**TABLE 4:** Dying, death and organ procurement. Domains and Quotes.

The pressure for organ donation success and its potential impact on end-of-life practices
Quote 1: “There is a form of pressure because we know the patient can donate his organs and save lives” (Nurse interview N03)
Quote 2: “The doctor in charge is caught between two injunctions: to ensure a dignified end of life for the patient, and to respect the deadlines imposed by the procedure” (Nurse interview N09)
Quote 3: “There’s this idea like… ‘hurry, he must die’” (Physician interview P08)
Quote 4: “There is a strong temptation to push what needs to be pushed in order to be within the deadlines” (Physician interview P03)
Quote 5: “I don’t feel comfortable with this possibility. Indeed we know that sometimes there is transgression” (Physician interview P02)
Procedural failures as a positive ethical signal
Quote 6: “I want things to go well so that the organs can go to people who need them and who can get better. That, for me, is a positive issue. If organs can’t be transplanted, well for me it’s a negative experience” (Physician interview P01)
Quote 7: “The institution puts a lot of pressure on us. We have to resist. We must accept that sometimes the procedure fails. We’re all convinced that the team will be more at ease with this activity if we screw up a situation once in a while” (Physician interview P03)
A modified experience of dying and death
Quote 8: “Family members don’t know where to put themselves, it’s complicated for us” (Physician interview P04)
Quote 9: “There were 15 of us in the room, and the patient was already halfway through the surgery before the cDCD procedure. On the one hand, there was the surgeons’ timeframe; they were practically in their sterile clothes with a scalpel in each hand, ready. And on the other, there were the family members and I could see that they weren’t able to say goodbye to their loved one because there were too many people in the room, there was no possible intimacy” (Physician interview P02)
Quote 10: “There was no care or support. It was really very technical. It wasn’t a peaceful or just a normal dying atmosphere at all. The patient died so it’s “OK he’s dead, that’s it, let’s start the clock” (Nurse interview N03)
Quote 11: “With everyone watching it’s just like a show. You want to say ‘come on, this isn’t a show, it’s a man dying’. I find it very difficult” (Physician interview P03)
Quote 12: “It all went well, technically it all went very well … But, in fact, we had forgotten that we were caring for a dying patient, as though he wasn’t there in a way” (Nurse interview N03)
Quote 13: “A patient who dies decently is just as important as a patient who heals” (Nurse interview N06)

### Ethical Tensions During the Decision-Making Process Leading to the Withdrawal of Life-Sustaining Treatments in a Context of Potential Organ Donation

#### A Gap Between Theory and Practice

In theory, the decision to withdraw LST should only be made in the patient’s best interest, must comply with the legal requirements, and should be independent of any subsequent consideration (including organ donation). However, in practice, physicians and nurses expressed their inability to set aside the potentiality of cDCD during the WLST decision-making process (Table 2, quote 1). This gap between theory and practice is experienced as a difficulty (quote 2).

#### Formal and Informal Communication

One strategy for dealing with this difficulty is to adopt a dual approach combining formal and informal communication (quote 3). Formal communication asserts official recommendations, namely the independence between WLST decision and organ donation possibility. For this purpose, a formal multidisciplinary meeting is organized by the medical team to explicitly reaffirm the priority of the patient’s best interest over the potentiality of organ donation. Field observation revealed that physicians set the scene in order to show to the other ICU staff members that organ donation has not been considered and that attention is focused solely on the WLST decision (quote 4). Physicians explained how, during the meeting, this dissociation between the WLST decision and the possibility of subsequent organ donation helps healthcare professionals to understand and accept the decision (quote 5). They also believed that it legitimated the WLST decision by removing doubt concerning a possible conflict of interest. In contrast, backstage informal communication allowed to consider organ donation as a possibility during the WLST decision-making process (quote 6).

#### End-of-Life Care as a Process, Organ Donation as a Procedure

Another strategy for dealing with this difficulty is one the hand to define end-of-life care as a process and an ethical priority and, on the other, to define organ donation as a strict procedure (quote 7).

#### Making Sense of the Ethical Dilemma

Participants perceived the gap between theory and practice as “impossible,” “hypocritical,” and “schizophrenic.” The ethical tension appeared to be partly resolved by considering organ donation as a way to give meaning to the patient’s death (quote 8). This consideration is not restricted to the patients themselves but is in fact extended to the future transplant recipients. This utilitarian approach allows healthcare professionals to consider cDCD in a broader benefit-risk balance (quote 9).

### Ethical Tensions During the Period Between the Decision to Withdraw Life-Sustaining Treatments and Its Actual Implementation

The tension between end-of-life care and organ donation is particularly evident during this period. Combining taking care of the patient during end of life and organizing the organ donation procedure, with its technical and operational requirements, can be challenging for healthcare professionals.

#### Experience of Dual Objectives: An Example From the Field Observation

A particular situation led to intense debates within the ICU team. A 36-year-old patient was identified as a potential cDCD donor. During the 48 h required to organize the cDCD procedure, he developed a heparin-induced thrombocytopenia with pulmonary embolism. The question for the team was how to deal with a potential worsening of the situation. Some members of the ICU team felt uncomfortable with this double objective: on the one hand providing end-of-life care and avoiding unnecessary treatments and, on the other hand, preserving the organs before they were retrieved. Each new complication that occurred during this period was an opportunity to discuss the tensions they experienced.

#### A Difficult Compromise Between End-of-Life Care and Organ Preservation

For half of the interviewed ICU staff members, the introduction or the increase of treatments that are no longer necessary for the patient but that are useful to preserve organ viability raises ethical questions and discomfort (Table 3, quotes 1 and 2). For the other half, and as in the situation described above, a compromise is possible and severe complications should be treated on two conditions: first they should not compromise the organ procurement proposal, and second the patient should be kept under deep and continuous sedation until death (quote 3).

#### A Time to Support Relatives

The participating nurses were adamant to use this time period to reword the physicians’ explanations and to provide emotional support to the relatives (quote 4). They insisted that special attention was given to the dying patient, which enables the organization of end-of-life rituals (quote 5). Last, this time period also allowed healthcare professionals and relatives to provide active verbal and non-verbal support to the patient, thus encouraging patient-centered care (quote 6).

### Ethical Tensions During Dying, Death, and Organ Procurement Procedure

French regulation specifies that, following WLST, the agonic phase—that is, the time running from treatment withdrawal to death—has to be less than 180 min in order to allow organ procurement.

#### The pressure for Organ Donation Success and Its Potential Impact on End-of-Life Practices

Participants reported increased stress during the implementation of decisions to withdraw LST (Table 4, quote 1), related to the fact that circulatory death must occur within the timeframe required for organ donation to be successful (quotes 2 and 3). This pressure on success can lead to changes in end-of-life practices, particularly regarding sedative practices (quote 4). This potential impact of the cDCD procedure on sedative practices is experienced as difficult for many healthcare professionals (quote 5).

#### Procedural Failures as a Positive Ethical Signal

A strategy for dealing with this pressure is to define a successful organ donation procedure as one that results in effective organ procurement (quote 6). However, another strategy exists to feel ethically comfortable: many physicians reported that they were reassured when a cDCD procedure failed because the patient didn’t die within the allowed timeframe. This procedural “failure” gives an opportunity to place the patient—rather than the organ donation—at the heart of their practice (quote7).

#### A Modified Experience of Dying and Death

The systematic use of normothermic regional perfusion offers logistic advantages to the relatives, especially the continuation of end-of-life care in the ICU. However, our field observations showed that end-of-life support was not always optimal and that he atmosphere in the room was deemed as being not appropriate for providing support (quote 8). This difficulty is even more acute when WLST occurs in the operating room where relatives are unable to support the patient and to say goodbye (quote 9). Several participants highlighted the fact that organ procurement is an exceptionally technical procedure (quote 10). Healthcare professionals sometimes take the opportunity to attend the procedure although they are not directly involved in the patient’s care, which was perceived as a form of voyeurism that may further desacralize the patient’s end of life (quote 11). Last, healthcare professionals often felt that they were unable to care for the dying patient as they would have liked to (quote 12). Hence, they felt that they were “stealing the patient’s death” from both the patient him/herself and from the relatives. This was problematic for healthcare professionals who described quality of dying as a major criterion for the quality of their work (quote 13).

## Discussion

**FIGURE 1:**
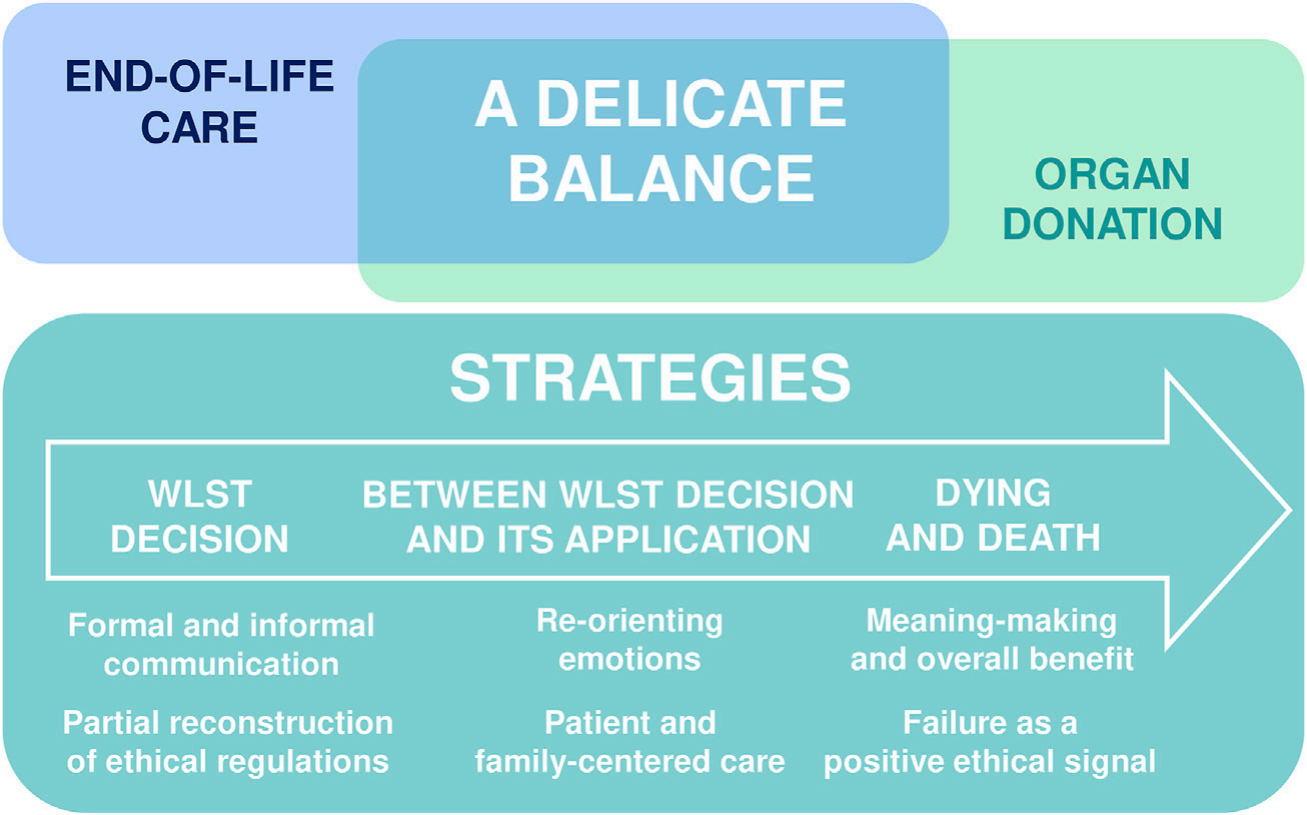
Experienced and perceived solutions and strategies.

National policies and guidelines have attempted to shape the process of cDCD into a routine activity for healthcare professionals so that it can become an accepted practice (15, 27). Ethical frameworks imply that healthcare professionals should not experience a moral tension between caring for the dying patient and altering his/her care for the purpose of donation. The interviews conducted during our study show that in practice the situation is more complex for both ICU physicians and nurses with a delicate balance between, on the one hand, end-of-life care and, on the other, organ donation (Figure 1). Indeed there is a gap between ethical theories and practice (28, 29) that clinicians seek to fill the best they can at all stages of the process.

Concerned simultaneously about end-of-life care and organ donation, healthcare professionals do not want to act against their moral principles and thus develop five types of strategies to solve the ethical and emotional tensions they experience (Figure 1). The first strategy used relies on virtue-centered communication (29). Physicians learn to be demonstrative by staging a distinct temporality between the WLST decision and the organ donation discussion in order to internalize the ethical principal at the basis of cDCD: the separation between WLST decision-making and organ donation decision-making. The second strategy is partial reconstruction of ethical regulations: once the demonstration described above has operated, clinicians can more openly express the intellectual and emotional limits of this practice. The third strategy concerns re-orientating emotions: at the time of WLST decision, some healthcare professionals focus on the WLST decision-making process by relegating organ donation to a secondary organizational and logistical issue. Once the decision to WLST has been made, healthcare professionals may experience important discomforts concerning end-of-life care vs. organ preservation strategies, or tensions concerning the direct exposure of relatives to the organizational dimensions of death. Instead of dwelling on the ethical tensions surrounding the patients’ treatment in anticipation of organ donation, they seek to use the extra time to provide quality support and care to the relatives and to ensure that healthcare professionals and relatives accept and adhere to both the WLST decision and the organ donation project. For some physicians and nurses, this delay may contribute to the quality of the patient’s death by allowing time for the relatives to be at the patient’s side and to say goodbye. The fourth strategy implies defending the principal of “overall benefit”. Indeed when confronted with death in the context of cDCD clinicians can experience moral distress and the feeling of “robbing” the patient’s death. To overcome this tension, the overall benefit of organ donation serves to maintain motivation. The fifth and last strategy implies necessary failures of the cDCD end-of-life procedure: ensuring that failure can happen (i.e., the patient doesn’t die within the timeframe) is a comfort for clinicians in that the quality of the person’s end-of-life takes precedence over the technical procedure.

One important finding of our study is that cDCD procedures are the result of several days of emotional and ethical tension between healthcare professionals, most often shared with the patient’s relatives. cDCD reshapes end of life in ICU, as end-of-life care is not only followed by death but also by organ donation. Despite the above-mentioned strategies, none of the stages of the process are black or white and there are no undisputable solutions to the complexity of the moral tensions experienced. Clinicians navigate in “grey areas,” juggling with official guidelines and ethical dilemmas, as well as with concrete moral, intellectual and emotional difficulties. Their task is to give meaning to the process, a meaning that can be shared with the patients’ family members and among the team (30). These concerns around “ethics in practice” take place within an ICU and, each time, within a specific ethical climate (31).

Healthcare professionals are vital for the implementation of cDCD and their attitudes can influence their participation. Satisfaction with end-of-life care impacts on physicians’ and nurses’ well-being (32) as well as on relatives’ well-being both during and after the patient’s death (33–35). Quality of communication between team members (36), adapted leadership and involvement of nurses (37) at all stages of the process are important elements that will help clinicians overcome these ethical tensions as a group—left alone to deal with these tensions, clinicians could develop moral distress and burnout leading to leaving the ICU (38).

Our study has some limitations. First, it was conducted in a single country (France), with specific end-of-life legislation (13) and cDCD protocols (15). Moreover, it was conducted in a single ICU, one of the first to have implemented this procedure, with a potential impact of the unit culture on the results. However, results of this exploratory single-centre study provide insights into healthcare professionals’ experience that may help design future multicentre studies (39). Last, although our purposive sampling strategy was designed to maximize the diversity of ICU clinicians who participated in the study, our results are—by definition—not entirely generalizable to all healthcare professionals working in ICU. Participation in qualitative interviews was voluntary, creating a possible selection bias: clinicians with difficulties in (or reluctance to) expressing themselves or their experiences concerning the cDCD process may have been omitted. Last, only one researcher coded the interviews. However, to reduce the risk of bias, two other researchers independently coded 3 transcripts for intercoder reliability that proved to be good. Any difficulties or uncertainties encountered by the main coder were discussed and resolved during team meetings.

This qualitative study provides in-depth understanding of the experience of ICU clinicians of the cDCD process. Despite clear and transparent national guidelines, the process remains entangled in a variety of ethical and emotional ambiguities that they strive to solve using various strategies. Overall, ICU clinicians believe that the implementation of cDCD is ethically reasonable as long as end-of-life care is preserved. Taken together, our results indicate that although national guidelines for cDCD are warranted to create a common legal, clinical and ethical framework, the implementation of cDCD in routine practice requires a shared understanding of the difficult compromises experienced by ICU clinicians between end-of life care and organ donation among ICU clinicians.

## Data Availability

The raw data supporting the conclusion of this article will be made available by the authors, without undue reservation.
