# Supplementary material for: “A Delicate balance”—Perceptions and Experiences of ICU Physicians and Nurses Regarding Controlled Donation After Circulatory Death. A Qualitative Study
Source: Transpl Int. 2022 Sep 6;35:10648. doi: 10.3389/ti.2022.10648 (PMC9485469; doi:10.3389/ti.2022.10648)
Supplement: Supplementary file 1 [file Table1.DOCX]

| **Supplemental Table 1. Semi-structured interview guide** | | |
| --- | --- | --- |
| **Themes** | **Objectives** | **Questions (examples)** |
| Introduction | Put the participant at ease. Identify the context and the participant’s characteristics | Can you introduce yourself? Tell me about your professional trajectory, its highlights, its turning points |
| General overview – cDCD as a practice | Introduce and focus the interview on cDCD | How would you explain the cDCD procedure? How does it work in practice? In your opinion, which patients are potentially concerned by this procedure? |
| Decision-making | Highlight the mechanisms of the decision-making process | Could you describe the decision-making process leading up to a cDD procedure? What makes a good decision-making process? What makes a bad one? What is your involvement/role in the decision-making process? How do you perceive the role of the organ donation team in the decision-making process? |
| Enactment of the WLST decision | Highlight medical and technical practices | Could you describe a patient’s end of life followed by a possible cDCD procedure? What is your role during the end of life process? What is your role regarding the cDCD procedure? Are you comfortable in this/these role(s)? What is your experience of the overall process? How much autonomy do you have in carrying out your actions? Have you experienced transgression in certain situations? |
| Interaction with the patient | Highlight interactions/relationships between HCPs and patients in this context | What is your relationship with the end-of-life ICU patient? Has this relationship evolved during your career? How would you describe your relationship with patients at the end of life before you were in a cDCD procedure? Does this relationship evolve with the cDCD procedure? |
| Interaction with the relatives | Highlight interactions/relationships between HCPs and relatives in this context | During this process, on what occasions do you interact with the patient’s relatives? Has this happened to you often? How do you experience these moments? How would you describe the relationship between healthcare professionals and relatives on a daily basis vs. during the cDCD procedure? In your opinion, what are the criteria for a healthy relationship with relatives in this context? |
| Experience:  ICU culture | Highlighting the ICU culture, ethical climate and communication between healthcare professionals | In your opinion, does the cDCD procedure have an impact (on practices, atmosphere, organisation, relationships, etc.) in the ICU? Are some beliefs unanimously shared or not? Are you able to exchange with your colleagues during or after the procedure? Does the ICU organise debriefing sessions? |
| Experience:  Personal beliefs and experiences | More intimate questions about participant’s experiences, emotions, difficulties and satisfactions | How do you personally feel about cDCD? What are your beliefs about end-of-life decision-making in this context? Organ donation? And cDCD? Do you have any difficulties? What solutions have you found? What have you learnt from these experiences? |
| Feedback from one specific cDCD experience | Identify specificities of EOL and organ donation in this context | Could you tell me about the last cDCD procedure you attended (decision-making etc.)? Do you remember the patient? Were his relatives present during his hospitalisation? During dying and death? Could you describe communication with them in this context? How do you think they experienced their loved-one’s end of life? Did you have any contact with them afterwards? |
| Conclusion and reflexion | Take stock of what has been said and the topics discussed. Give the respondent the opportunity to correct himself and add spontaneous elements. | We discussed... For you, it appears that... Is there anything you would like to add or take back? What did you think of this interview? |
